# Phenome-wide and expression quantitative trait locus associations of coronavirus disease 2019 genetic risk loci

**DOI:** 10.1016/j.isci.2021.102550

**Published:** 2021-05-18

**Authors:** Chang Yoon Moon, Brian M. Schilder, Towfique Raj, Kuan-lin Huang

**Affiliations:** 1 Department of Genetics and Genomic Sciences, Icahn School of Medicine at Mount Sinai, New York, NY 10029, USA; 2 Center for Transformative Disease Modeling, Icahn School of Medicine at Mount Sinai, New York, NY 10029, USA; 3 Tisch Cancer Institute, Icahn School of Medicine at Mount Sinai, New York, NY 10029, USA; 4 Icahn Institute for Data Science and Genomic Technology, Icahn School of Medicine at Mount Sinai, New York, NY 10029, USA; 5 Nash Family Department of Neuroscience & Friedman Brain Institute, New York, NY, USA; 6 Ronald M. Loeb Center for Alzheimer's disease, Icahn School of Medicine at Mount Sinai, New York, NY, USA; 7 Estelle and Daniel Maggin Department of Neurology, Icahn School of Medicine at Mount Sinai, New York, NY, USA

**Keywords:** Risk factor, Virology, Omics, Genomics

## Abstract

While several genes and clinical traits have been associated with higher risk of severe coronavirus disease 2019 (COVID-19), how host genetic variants may interact with these parameters and contribute to severe disease is still unclear. Herein, we performed phenome-wide association study, tissue and immune-cell-specific expression quantitative trait locus (eQTL)/splicing quantitative trait locus, and colocalization analyses for genetic risk loci suggestively associated with severe COVID-19 with respiratory failure. Thirteen phenotypes/traits were associated with the severe COVID-19-associated loci at the genome-wide significance threshold, including monocyte counts, fat metabolism traits, and fibrotic idiopathic interstitial pneumonia. In addition, we identified tissue and immune subtype-specific eQTL associations affecting 48 genes, including several ones that may directly impact host immune responses, colocalized with the severe COVID-19 genome-wide association study associations, and showed altered expression in single-cell transcriptomes. Collectively, our work demonstrates that host genetic variations associated with multiple genes and traits show genetic pleiotropy with severe COVID-19 and may inform disease etiology.

## Highlights

• Immune, metabolic, and respiratory traits are associated with severe COVID-19 loci
• Tissue- and immune-subtype-specific eQTL analyses uncover associated immune genes
• Immune gene eQTLs colocalize with COVID-19 GWAS associations
• Macrophages of patients with severe COVID-19 differentially express eQTL-linked genes

## Graphical abstract

**Figure:**
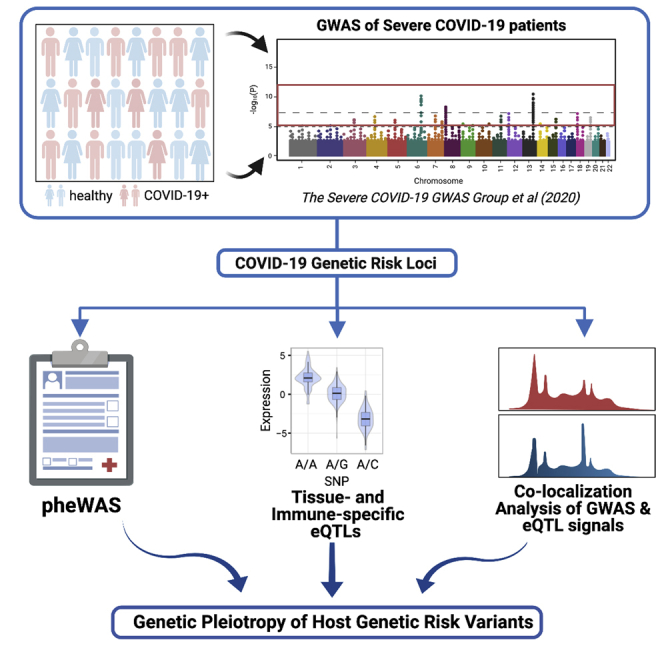

## Introduction

Upon emerging from Wuhan, China, in December 2019, the outbreak of coronavirus disease 2019 (COVID-19), caused by severe acute respiratory syndrome coronavirus-2 (SARS-CoV-2), has become a global pandemic, infecting, as of August 2020, more than 24 million people worldwide (Dong et al., 2020) While most infected individuals either are asymptomatic or present with mild flu-like symptoms (i.e., fever, cough, sore throat, malaise, and so on), few experience severe manifestations of COVID-19 that span multiple organ systems (Chen et al., 2020; Guan et al., 2020; Huang et al., 2020). Although several clinical observational studies have identified several demographic and clinical factors associated with increased disease severity and mortality such as sex, age, and other comorbidities such as cancer, chronic kidney disease, and obesity, which and how host genetic factors may contribute to the pathogenesis and pathophysiology of severe COVID-19 are poorly understood (Williamson et al., 2020) While a recent effort by the severe COVID-19 genome-wide association study (GWAS) group identified two significant loci (3p21.31 and 9q34.2) and multiple suggestive host genetic loci associated with increased risk of suffering from severe COVID-19 with respiratory failure, the functional effects of these loci and how they may influence disease severity remain unclear (Ellinghaus et al., 2020).

A phenome-wide association study (PheWAS) is a genotype-to-phenotype approach to identify associations between a particular genetic variant and a wide range of human traits (Hebbring, 2014). Since the first published PheWAS in 2010(Denny et al., 2010), the PheWAS has been used to interrogate genetic pleiotropy, facilitate drug discovery and repositioning and discover novel genetic etiologies of complex and polygenic disorders (Diogo et al., 2018; Hebbring, 2014; Semmes et al., 2020; Zhao et al., 2018) and systematic compilations of GWAS results, such as that recently undertaken by GWAS Atlas, have facilitated the PheWAS (Watanabe et al., 2019). Considering the multiorgan involvement and manifold individual variations of severe COVID-19, it is likely that host susceptibility to severe COVID-19 is determined by multiple genetic factors. By connecting variants associated with both severe COVID-19 and other diseases or phenotypes, PheWAS analysis can use genetic pleiotropy to validate known comorbidities and unveil novel biological insights. Furthermore, PheWAS may offer new insights into the genetic etiology of severe COVID-19 by leveraging known biology behind seemingly disparate phenotypes.

In addition to PheWAS that can link categorical phenotypes, tissue-specific quantitative trait locus (QTL) analysis can help to prioritize gene-disease connections for mechanistic investigations. With the advent of immense functional genomic databases such as GTEx (GTEx Consortium et al., 2020), the transcriptional effects of genetic variants can be investigated at tissue-specific resolution. The findings gathered from tissue-specific QTL analysis to severe COVID-19 can help to (1) identify candidate genes that may contribute to the pathophysiology of severe COVID-19, (2) focus the range of mechanistic hypotheses regarding the effect of specific gene expressions to organs most affected by severe COVID-19, and (3) cross-reference with known biology of linked phenotypes uncovered by the PheWAS analysis to validate and refine hypotheses regarding the pathophysiological mechanisms of severe COVID-19.

In this present study, we conducted both PheWAS and QTL analyses to identify potential mechanisms through which host genomic factors contribute to the pathogenesis and pathophysiology of severe COVID-19. We extracted the lead single-nucleotide polymorphism (SNP) with the smallest p value from each locus showing suggestive associations with severe COVID-19 cases with respiratory failure using the recent GWAS (Ellinghaus et al., 2020) and conducted the PheWAS using these lead SNPs by leveraging the GWAS Atlas database and identified a list of linked phenotypes. We systematically performed colocalization analyses between each locus identified by the PheWAS and 112 QTL data sets from GTEx (GTEx Consortium et al., 2020) and the eQTL Catalogue (Kerimov et al., 2020), spanning 69 unique tissue types. This provided a robust list of candidate genes with their tissue-specific expression alterations. Together, the results presented here have generated multiple focused hypotheses regarding the pathophysiology of severe COVID-19 for future validation and investigation.

## Results

### PheWAS of genetic risk loci associated with severe COVID-19

To identify phenotypes sharing genetic etiologies with severe COVID-19, we conducted PheWAS of 22 pruned genetic risk loci significantly or suggestively associated (p < 1e-5) with severe COVID-19 based on GWAS results published by The severe Covid-19 GWAS group (accession numbers: GCST90000256) (Ellinghaus et al., 2020) (Table S1). We first sought to replicate the 22 SNPs using GWAS performed by the COVID-19 Host Genetics Initiative (COVID-19 hg). Five SNPs, including rs2440652, rs3934992, rs12610495, rs11385942, and rs657152, showed an association passing the Bonferroni threshold (p < 0.05/22) and were further considered as “COVID-19 hg-replicated SNPs” in the following texts. To gauge the independence of the signal for each lead SNP, linkage disequilibrium (LD) analysis was performed using the LDmatrix tool (Machiela and Chanock, 2015). LD analysis demonstrated that the 22 lead SNPs were independent from each other (R^2^ < 0.01; Table S2) and each replicated SNP was found within an LD block (R^2^ > 0.8) consisting of its neighboring variants.

**Figure 1:**
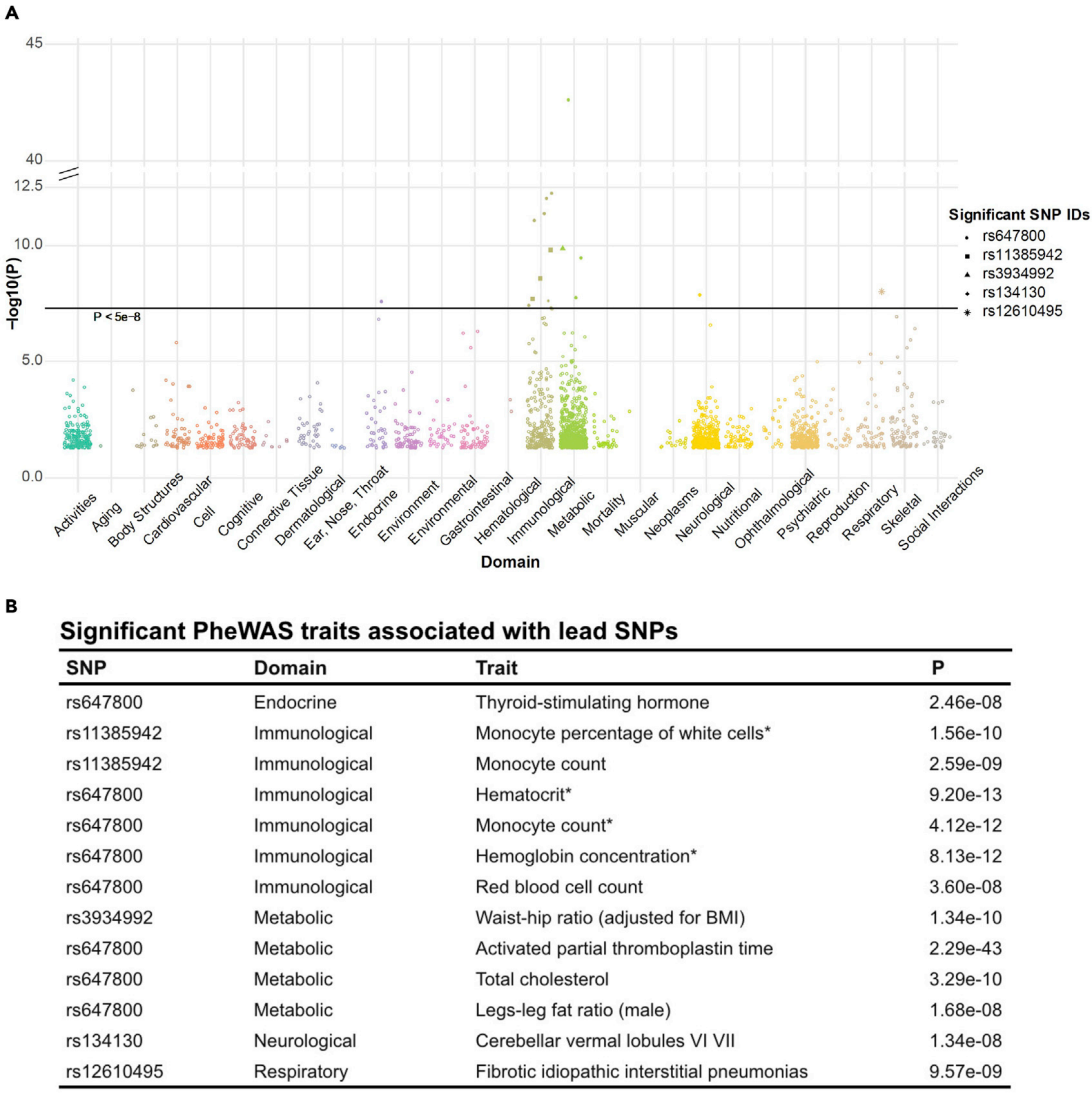
PheWAS of the lead SNPs showing suggestive associations (p < 1e-5) with severe COVID-19 (A) A Manhattan plot of the phenotypes associated with the lead SNPs. Only phenotypes that had p-values reaching genome-wide significance (p < 5e-8, marked by the horizontal line) were marked with their associated SNPs (See “Significant SNP IDs”). (B) A table of significant associated traits. (∗) indicates that there were duplicate entries resulted from two- and three-way meta-analyses for the same trait, where the three-way meta-analysis results were used for all these associations. See also Figure S1 and Tables S1, S2, and S3.

On conducting the PheWAS of the 22 lead SNPs using GWAS Atlas (Watanabe et al., 2019) that has aggregated summary statistics of more than 4,756 GWAS studies, we identified 5 SNPs, including the 2 reported COVID-19 GWAS SNPs (rs647800, rs11385942)(Ellinghaus et al., 2020) and two COVID-19 hg-replicated SNPs (rs3934992, rs12610495), that showed associated traits reaching the genome-wide significance threshold (p < 5e-8) (a full list of PheWAS results was listed in Table S3). A total of 13 traits were significant (Figure 1A)—8 of which were associated with rs647800, an intronic variant of the *ABO* gene (Yeung et al., 2017). Significant associated traits were predominantly immunological or metabolic (Figure 1B). The immunological traits were mostly relevant to population counts/proportions of monocytes and erythrocytes. Top metabolic traits were biochemical and physiological metrics concerning coagulation (“activated partial thromboplastin time”) and fat metabolism (“total cholesterol,” “legs-leg fat ratio [male]). Of all the significantly associated traits, fibrotic idiopathic interstitial pneumonia was the only disease linked through the COVID-19 GWAS-lead SNPs. Taken together, the PheWAS analysis of 22 lead SNPs identified several physiological metrics as well as a disease associated with severe COVID-19.

### eQTL/sQTL analyses

**Figure 2:**
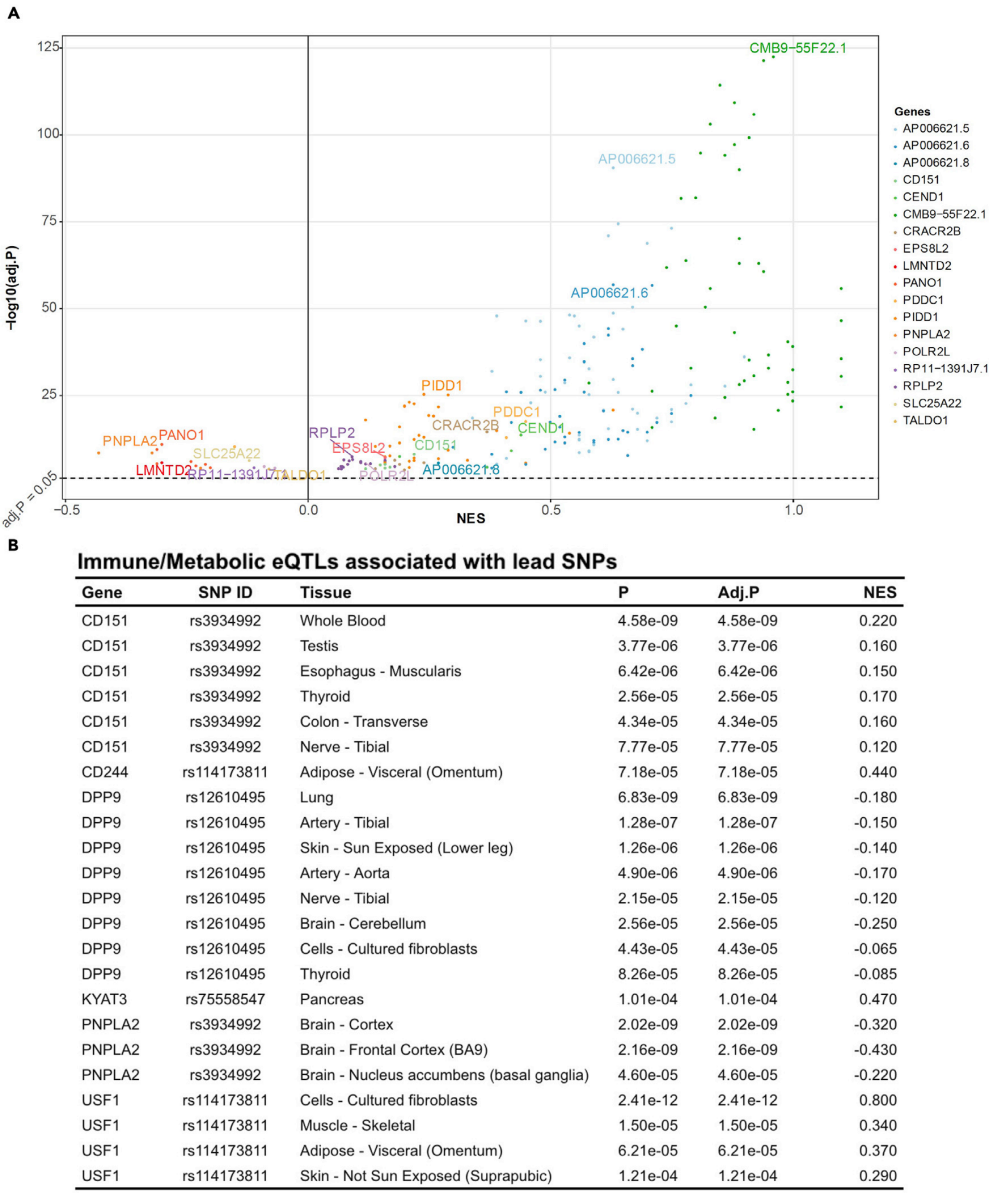
Significant eQTLs associated with lead SNPs (A) A volcano plot of eQTLs associated with rs3934992. Dashed line (-) represents adjusted p-value of 0.05. NES: Normalized Effect Size. (B) A table of eQTLs affecting immune and metabolic genes. See also Tables S4 and S5

To evaluate the functional relevance of the lead GWAS loci, we used 22 lead SNPs to query GTEx and compiled e/sQTLs associated with the lead SNPs. After filtering associations based on QTL FDR < 0.05, 11 lead GWAS SNPs were identified as significant e/sQTLs (Tables S4 and S5) with the vast majority of eQTLs (n = 227 of 258) and sQTLs (n = 140 of 143) being associated with 1 SNP (rs3934992; Figure 2A). No one particular tissue type dominated the eQTL and sQTL associations; the top tissue types with associated eQTLs include the muscularis layer of the esophagus (n = 10), tibial nerve (n = 10), sun-exposed skin at the lower leg (n = 10), and lungs (n=6). The eQTL analysis encompassing 22 lead SNPs identified 29 novel genes in addition to 4 genes (i.e., *SLC6A20*, *FYCO1*, *CXCR6*, *CCR1*) previously reported by the severe COVID-19 GWAS group to be associated with rs11385942 at locus 3p21.31 (Ellinghaus et al., 2020). Two sets of three genes (*CMB9-55F22.1*, *PIDD1*, *RPLP2*), all of which are associated with the predominant lead COVID-19 hg-replicated SNP (rs3934992), constituted top 10% of statistically significant eGenes and sGenes, respectively.

In addition to these top genes, we also identified several genes with known immune and metabolic functions—paralleling our findings in the PheWAS analysis (Figure 2B). Corresponding to the top metabolic PheWAS traits related to fat metabolism, one distinct COVID-19 hg-replicated SNP was a genome-wide significant e/sQTL of *PNPLA2*, a critical enzyme involved in the first step of triglyceride hydrolysis (Taxiarchis et al., 2019). In terms of immunity, expression changes in *CD151*, *PIDD1,* and *DPP9*—genes involved in immune cell activation and NF-kB-mediated immune response (Okondo et al., 2017; Rocha-Perugini et al., 2014; Tinel et al., 2007)—were associated with two distinct COVID-19 hg-replicated SNPs (rs3934992, rs12610495). Overall, QTL analyses of the lead SNPs identified genes of which expression changes may be functionally associated with severe COVID-19 at a tissue-specific resolution.

### Immune-specific eQTL analysis

**Figure 3:**
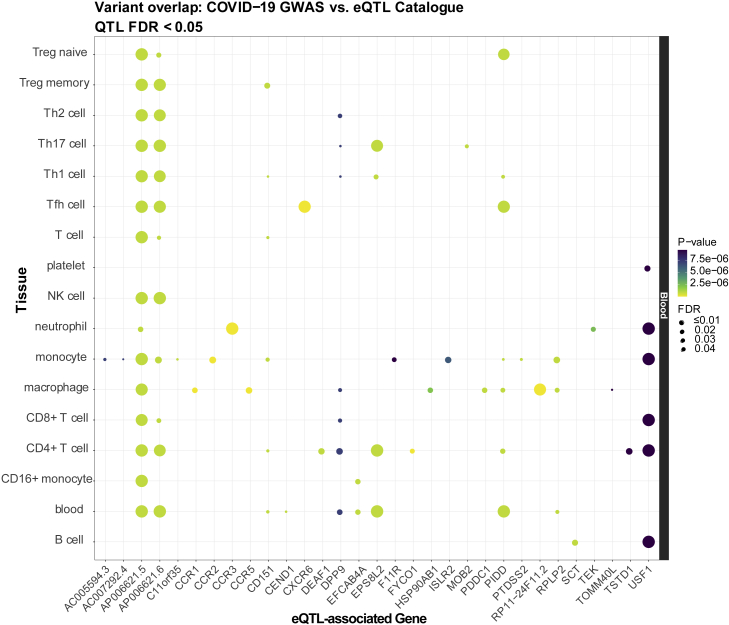
Significant immune subtype-specific eQTLs associated with lead SNPs The eQTLs were compiled from the eQTL Catalogue (Kerimov et al., 2020) database and filtered at a Benjamini-Hochberg FDR < 0.05. Point color reflects the significance of that SNP in the COVID-19 GWAS (Ellinghaus et al., 2020) (more yellow = more significant p-values), while point size reflects the significance of the eQTL SNP (larger = more significant FDR). When multiple SNPs overlap within a given gene-tissue combination, the SNP with the lowest p-value/FDR is plotted. See also Figures S2 and S3 and Table S6

Based on the identification of genes with known immune function in the eQTL analysis, as well as the reported involvement of the dysregulated immune response in severe COVID-19 (Lucas et al., 2020), we further queried the 22 lead SNPs in the eQTL Catalogue to determine whether they affected gene expression in specific immune cell subtypes. We identified 134 immune-cell-subtype-specific eQTL associations (FDR <0.05) that are distributed across 7 GWAS SNPs and 31 eGenes. Several genes that were the most statistically significant in the GTEx analysis (*RPLP2*, *PIDD1*) reemerged in the immune-subtype-specific eQTL analysis and were primarily seen in monocytes and macrophages (Mo/Mφ) and several T cell subsets. Moreover, expression changes of a few immune genes (*DPP9*, *CD151*, *PIDD1*) that were previously highlighted were predominantly seen in both CD4^+^ and CD8^+^ T cells as well as Mo/Mφ. Notably, 14 of these genes were not nominated by the GTEx eQTL analysis but were associated in various T, B, natural killer (NK), and myeloid cell subsets (Figure 3), including several genes involved in leukocyte recruitment and migration such as *CCR1*, *CCR2*, *CCR3*, *CCR5,* and *CXCR6*. These results highlight the resolution gained by conducting cell-type-specific analyses into immune-cell-expressed genes that may be linked to the genetic factors underlying severe COVID-19.

### GWAS/eQTL colocalization analysis

**Figure 4:**
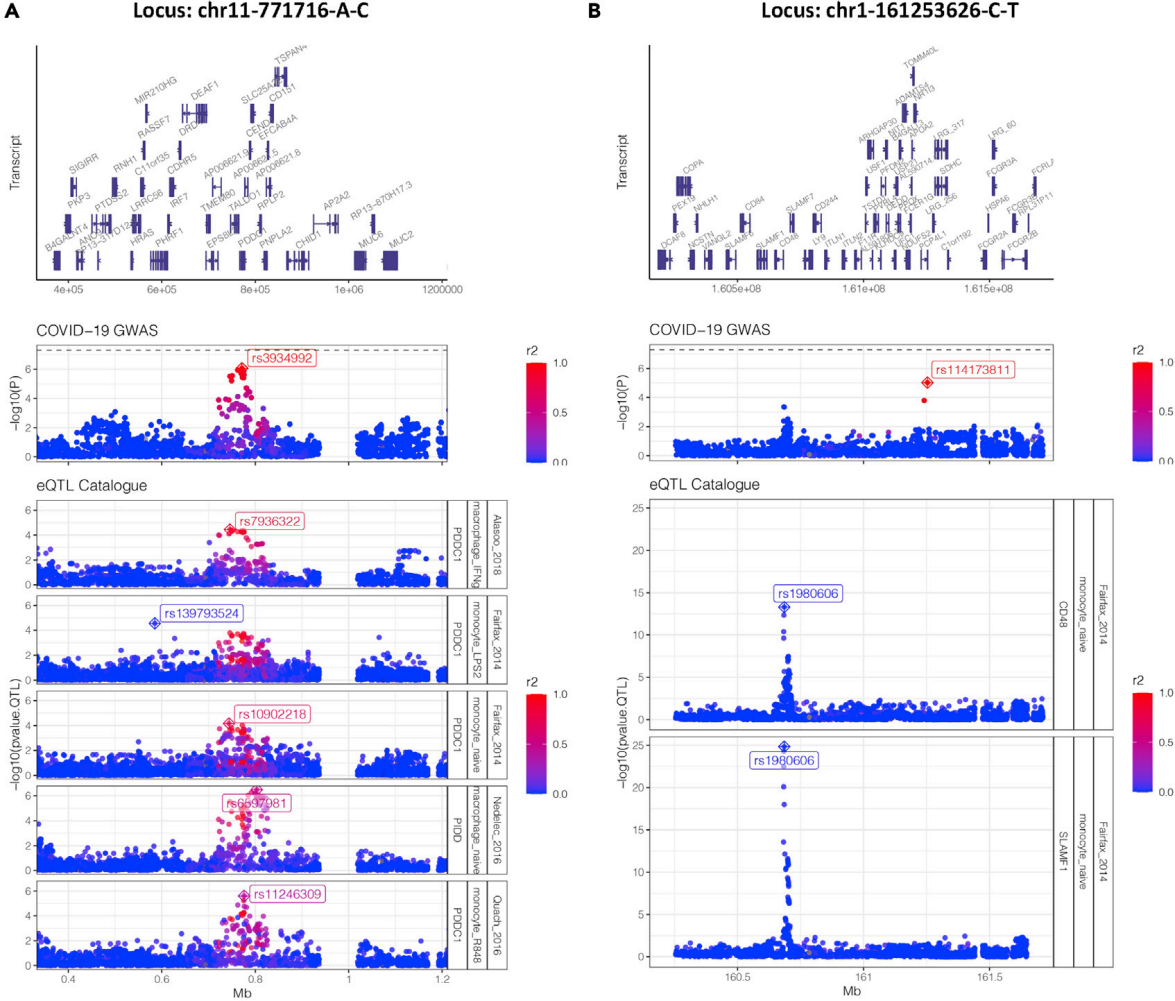
Colocalization analysis Manhattan plots of two severe COVID-19 GWAS loci (A) chr11-771716-A-C (B) chr1-161253626-C-T and their respective colocalized immune eQTLs from the eQTL Catalogue (colocalization probability >0.8). Each point represents an SNP across a genomic range, and color indicates the degree of LD (r^2^) with the proxy GWAS SNPs in each locus (indicated by crossed diamonds). In the eQTL rows, crossed diamonds indicate the lead QTL SNP (with the smallest association p value for a given gene).

Owing to LD, overlapping significant SNPs between GWAS and QTL do not necessarily mean they share the same underlying causal signal. Or does a lack of overlapping lead variants necessarily mean that they do not share the same signal being tagged by different variants. To robustly test for GWAS-eQTL signal sharing, we performed a series of colocalization analyses (Wakefield, 2009) in the 9 loci in which the GWAS-lead SNPs overlapped with significant eVariants (FDR <0.05) in the eQTL analysis. We identified 7 colocalizations that met the posterior probability threshold (see STAR Methods), spread across 2 GWAS loci, 5 eQTL data sets (all in macrophages and monocytes), and affecting the expression of 4 genes (*PDDC1*, *PIDD1*, *CD48,* and *SLAMF1*) (Figure 4)

In locus chr11-771716-A-C (Figure 4A), the colocalized genetic signal both increased severe COVID-19 risk and modulated expression of *PDDC1* in monocytes under three conditions (naive, LP2, and R848) and in IFN-γ-stimulated macrophages. The expression of P53-induced death domain protein 1 (*PIDD1)* was also affected in macrophages (naive), suggesting that both *PDDC1* and *PIDD1* are perhaps controlled by a common regulatory element that is disrupted by the severe COVID-19 risk signal in this region.

While locus chr1-161253626-C-T was also colocalized with eQTLs in monocytes (naive), the signal modulated expression of *CD48* and signaling lymphocytic activation molecule family member 1*,* both of which are immunoglobulin-like receptors of the CD2 subfamily (Figure 4B). Interestingly, a closer inspection revealed that the colocalizing GWAS signal was not the same one tagged by the previously identified proxy SNP showing the most significant association with severe COVID-19 (rs114173811) but instead a signal ~0.5Mb upstream tagged by the SNP rs1980606. LD analysis confirmed that these signals are independent (no SNPs in the upstream signal had r^2^ > 0 with the lead SNP in the downstream signal). This highlights the complexity of genetic association signals as well as the additional insights that colocalization analyses can reveal.

### Differential gene expression in bronchoalveolar lavage fluid using single-cell analyses

To determine if expression of any of these eQTL-nominated immune genes as well as genes involved in implicated pathways (i.e., inflammasome activation) was altered in patients with severe COVID-19 compared with patients with moderate COVID-19 or healthy controls, we performed a DGE analysis on a recentlypublished scRNA-seq data set of bronchoalveolar lavage fluid of patients with COVID-19 of varying disease severity and healthy controls (Liao et al., 2020b). Because the functions of these immune genes are either most well-characterized in Mo/Mφs (*DPP9, NLRP1, GSDMD*) (Okondo et al., 2017) or known to be most relevant for Mo/Mφs (*CCR1, CCR2, CCR5*)(Dyer et al., 2019), we specifically focused our DGE analysis on Mo/Mφs subsets within the data set and evaluated the differential expression of six eQTL-nominated immune genes. Average expression of genes involved in inflammasome activation (*DPP9, NLRP1, GSDMD*) was significantly altered (adj. p < 0.001) between Mo/Mφs of patients with severe and moderate COVID-19. Moreover, average expression of several genes involved in leukocyte recruitment and migration (*CCR1, CCR2, CCR5*) was significantly higher (Bonferroni adj. p < 0.001) in Mo/Mφs of patients with severe COVID-19 than in both patients with moderate COVID-19 as well as healthy controls (Figure S3). These results demonstrate, in at least a subset of patients with COVID-19, the differential expression of eQTL-nominated immune genes between Mo/Mφs of patients with severe COVID-19 and of patients with moderate COVID-19 or healthy controls.

## Discussion

In this study, we characterized the phenome- and transcriptome-wide associations of genetic loci implicated in severe COVID-19 with respiratory failure. We leveraged GWAS ATLAS database (Watanabe et al., 2019) containing summary statistics from 4,756 GWASs to perform a PheWAS of the lead SNPs and identified 13 significant traits linked to genetic risk loci of severe COVID-19. Using both eQTL databases, we performed eQTL analysis of the lead SNPs to characterize their effects on gene expression in tissue- and immune-cell-subset-specific resolution. Eighteen lead SNPs were identified as eQTLs/sQTLs of 48 genes. Taken together, these results reveal insights into potential mechanisms that could drive the formation of focused hypotheses about the pathophysiology of severe COVID-19.

Immunological and metabolic traits were among significant phenotypes uncovered by the PheWAS analysis. Severe cases of COVID-19 are often characterized by dysregulated and hyperinflammatory immune system (Lucas et al., 2020; Merad and Martin, 2020; Vabret et al., 2020) and metabolic dysfunction (Ayres, 2020)—suggesting that these SNPs may either play a role in multisystem manifestations of severe COVID-19 or contribute to the development of comorbidities known to exacerbate the severity of disease. For instance, that top immunological traits were hematologic parameters such as monocyte and erythrocyte populations is concordant with the hematological findings in severe COVID-19 cases such as monocyte expansion (Merad and Martin, 2020; Wen et al., 2020) and decrease in hemoglobin levels (Chowdhury and Anwar, 2020; Li et al., 2020). Recent reports have suggested that cytokine profiles of patients with severe COVID-19 show similarities to cytokine release syndromes such as macrophage activation syndromes (Giamarellos-Bourboulis et al., 2020; Mehta et al., 2020; Moore and June, 2020) and that monocytes/macrophages extensively accumulate in the lungs (Sánchez-Cerrillo et al., 2020). Further investigations are required to disseminate how these lead SNPs (rs647800 and rs11385492) may similarly or differentially contribute to monocyte count and monocyte-driven inflammation seen in COVID-19.

Coupled with hyperinflammation driven partly by these mononuclear cells, another major prong of severe COVID-19 pathology is systemic coagulopathy. Patients suffering from severe COVID-19 often have lower levels of platelet counts and increased levels of D-dimers (Becker, 2020; Chan and Weitz, 2020; Iba et al., 2020; Liao et al., 2020a). Numerous cases of widespread pulmonary thrombosis and disseminated intravascular coagulation or sepsis-induced coagulopathy have also been described in severe COVID-19 patients (Becker, 2020; Tang et al., 2020). Possibly reflective of the predominant role of coagulopathies in the pathophysiology of severe COVID-19, the most statistically significant trait identified in our PheWAS analysis was activated partial thrombin time (p value = 2.29e-43; associated SNP: rs647800) which has been reported to be prolonged in patients with severe COVID-19. Notably, a recent review by Merad et al. (Merad and Martin, 2020) has suggested a link between hyperinflammatory monocytes and coagulopathies—as even in the absence of any vascular injury, coagulation pathway can still be activated through the recruitment of tissue-factor-expressing inflammatory monocytes. Taken together, it would be interesting to interrogate the genetic pleiotropy underlying rs647800 and how it affects monocyte count, activated partial thrombin time, and severe COVID-19.

In addition to coagulation, another metabolic traits highlighted by the PheWAS results were regarding cholesterol levels (“total cholesterol”; p-value = 3.29e-10) and physiological measures associated with obesity (“waist-hip ratio” and “legs-leg fat ratio”; p-values = 1.34e-10 and 1.68e-8, respectively). Both obesity and high cholesterol levels have been associated with increased risk of severe COVID-19 (Wang et al., 2020; Zhu et al., 2020), thereby suggesting that the PheWAS analysis can capture some of the comorbidities found by epidemiological studies.

The PheWAS also identified rs12610495 to be associated with fibrotic idiopathic interstitial pneumonias (FIIP; p-value: 9.57e-9), a disease directly connected to respiratory failure. FIIP shares a similar risk profile with severe COVID-19 disease and thus is a potential comorbidity of severe COVID-19 (George et al., 2020). Notably, in addition to sharing a similar risk profile with FIIP, some patients with severe COVID-19 suffer from lung function abnormalities that strongly suggest the presence of pulmonary fibrosis (Raghu and Wilson, 2020), implicating similar etiologies underlying COVID-19 and FIIP.

In line with the immunological and metabolic traits that emerged from the PheWAS analysis, our QTL analyses identified several genes with related functions that contribute to those traits. For instance, we found that rs12610495, an intron variant of *DPP9,* is within a susceptibility locus for FIIP (Fingerlin et al., 2013) and modulates both the expression and splicing of *DPP9. DPP9* can inhibit *NLRP1* and repress downstream inflammasome activation (Zhong et al., 2018). Conversely, pharmacological inhibition of *DPP9* has been shown to induce *NLRP1* inflammasome-mediated pyroptosis in monocytes and macrophages (Gai et al., 2019; Okondo et al., 2017; Vasconcelos et al., 2019; Zhong et al., 2018). *NLRP1*-dependent pyroptosis in the lung alveolar macrophages has been shown to cause progressive lung damage that is similar to the one seen in acute respiratory distress syndrome (Kovarova et al., 2012). Furthermore, *NLRP1*-dependent pyroptosis is sufficient to cause progressive lung injury, independent of *NLRP3* activation or *IL-1b* secretion, which suggests a relevant role of *DPP9* in mediating lung injury seen in severe COVID-19 (Kovarova et al., 2012). Finally, it was recently shown that human *NLRP1* senses double-stranded RNA (dsRNA) and activates an inflammasome complex upon dsRNA sensing (Bauernfried et al., 2021)—thereby raising a distinct possibility that dsRNA replication intermediates of SARS-CoV-2 (V’kovski et al., 2021) can be sensed by NLRP1 and set off an inflammatory cascade mediated by inflammasome activation. Considering the evidence together, it is possible that decreased *DPP9* expression in the lungs can predispose patients with COVID-19 to develop lung injury mediated by increased *NLRP1*-dependent pyroptosis. Concordant with this hypothesis, the lead SNP rs12610495 is associated not only with decreased expression of *DPP9* specifically in the lung and in macrophages but also with increased risk of severe COVID-19 with respiratory failure. Furthermore, our DEG analysis of scRNA-seq of bronchoalveolar fluid of patients with COVID-19 showed that expression of *DPP9, NLRP1,* and *GSDMD,* an effector protein for pyroptosis (Mathur et al., 2018), was significantly altered in Mo/Mφs of patients with severe COVID-19 compared to their moderate counterparts. Overall, our results suggest the potential role of inflammasome activation and pyroptosis in exacerbating respiratory distress in severe COVID-19—a point that was recently echoed in a recent review of inflammasomes in COVID-19 (Yap et al., 2020).

Beyond *DPP9*, our QTL analyses identified several immune genes that may play a role in potential mechanisms through which host immune response against SARS-CoV-2 can be affected. These potential mechanisms include NF-kB-mediated immune response (i.e. *PIDD1*) (Bock et al., 2013; Tinel et al., 2007), immune synaptic interaction involved in T cell activation (i.e. *CD151*) (Rocha-Perugini et al., 2014) and T cell exhaustion in viral infection (i.e. *CD244*) (Agresta et al., 2018; Pacheco et al., 2013). In our immune subset-specific QTL analysis, we were able to resolve changes in expression of *PIDD1* and *CD151* in diverse T cell subsets as well as monocytes. Moreover, the QTL overlap and colocalization analyses showed that the genetic signal associated with increased severe COVID-19 risk also modulated immune genes (i.e., *PDDC1*, *CD150, CD48*) in monocytes. For instance, one of the colocalized genes, *CD48*, is known to interact with *CD244* to modulate NK cell and CD8^+^ T cell effector function (Agresta et al., 2018; McArdel et al., 2016). Taken together, both QTL overlap and colocalization analyses highlight strong associations between risk of severe COVID-19 and transcriptional changes that underpin monocyte-lymphocyte interactions.

Furthermore, we also identified several leukocyte adhesion and chemokine receptors (*CCR3*, *CCR5*) in addition to the chemokine receptors (*CCR1*, *CCR2*, *CXCR6*) previously reported by the severe COVID-19 GWAS group (Ellinghaus et al., 2020)—all of which can mediate the increased leukocyte recruitment seen in the lungs of patients with severe COVID-19 (Sánchez-Cerrillo et al., 2020) In line with our immune-subset-specific QTL analysis and the aforementioned histological findings, we observed in our DEG analysis a significant increase in the expression of these chemokine receptors (*CCR1, CCR2, CCR5)* in Mo/Mφs of patients with severe COVID-19 compared with those of both patients with moderate COVID-19 and healthy controls. While current scRNA-seq data set did not include genotype information, future single-cell data sets with paired DNA/RNA data can help confirm the link between genetic risk allele and their eQTL effects at a single-cell resolution.

Concordant with several metabolic traits identified in the PheWAS such as obesity and cholesterol levels (Kanai et al., 2018; Pulit et al., 2019; Rask-Andersen et al., 2019), the QTL analyses identified two genes, *PNPLA2* and *USF1*, whose functions are directly connected to those traits. Furthermore, we were able to resolve changes in expression of *USF1* to the level of various immune cell compartments and found that *USF1* expression was altered predominantly in Mo/Mφs and T cell subtypes. *USF1* is a transcription factor that modulates several genes involved in lipid and glucose metabolism such as apolipoproteins and lipases including *PNPLA2*, a triglyceride lipase that catalyzes the initial step in triglyceride hydrolysis (Aguilar-Salinas et al., 2014; Taghizadeh et al., 2019). Knockout studies of *USF1* in mice have been shown to enhance cholesterol efflux in macrophages and downregulate secretion of pro-inflammatory cytokines such as MCP-1 and IL-1β (Ruuth et al., 2018) and *USF1* variants in humans have been associated with familial combined hyperlipidemia and coronary artery disease (Fan et al., 2014; Laurila et al., 2016; Ruuth et al., 2018). Interestingly, a recent study by Dias et al. showed that monocytes from COVID-19 have increased lipid droplet accumulation and that pharmacological inhibition of lipid droplet formation led to a significant inhibition of replication of SARS-CoV-2 (Dias et al., 2020). In addition to lipid metabolism, kynurenine metabolism has also been implicated by one of our nominated genes, *KYAT3*, an aminotransferase that transaminates kynurenine to kynurenic acid. Considering that kynurenine pathway plays an integral role in immunosuppression (Routy et al., 2016; Wirthgen et al., 2018; Zhai et al., 2020), it would be interesting to investigate how expression changes in *KYAT3* can affect the immune response against SARS-CoV2. Notably, alterations in both metabolic pathways implicated in the QTL analyses—fatty acid and kynurenine metabolism—have also been observed in patients with COVID-19 (Thomas et al., 2020), further supporting that these genes may play a role in immune dysfunction seen in severe COVID-19.

In summary, we conducted PheWAS and QTL analyses to investigate how host genetic factors may contribute to pathophysiology of severe COVID-19 and uncovered numerous gene-disease-tissue connections. Our results provide plausible consequences of the host genetic risk factors underlying severe disease, informing future investigations into immune, metabolic, and other aspects of COVID-19 pathophysiology.

### Limitations of the study

We acknowledge the limitations of our approaches, such as the restriction of discovering traits studied by available GWAS or QTL data sets and their correlational nature, which may be addressed by techniques such as Mendelian randomization as the COVID-19 GWAS expands in size (T.C.-19 H.G., 2020). Widespread LD also poses challenges when trying to distinguish causal variants from their close correlates—an issue which fine-mapping techniques aim to resolve (Broekema et al., 2020; Hutchinson et al., 2020; Schaid et al., 2018). Furthermore, the suggestive genetic risk loci of severe COVID-19 used for analyses herein as well as the identified PheWAS and QTL associations also require validation.

## STAR★Methods

### Key resources table

**Table:** 

REAGENT or RESOURCE	SOURCE	IDENTIFIER
**Deposited data**		

Genome-wide association study of patients with severe COVID-19	The severe COVID-19 GWAS group et al., PMID: 32558485	Accession number: GCST90000256
scRNA-seq of bronchoalveolar lavage fluid of COVID-19 patients	Liao et al.2020a, 2020bPMID: 32398875	Accession Number: GSE145926
GWAS Atlas	Watanabe et al., 2019PMID: 31427789	N/A
eQTL Catalogue	Kerimov et al., 2020(https://www.biorxiv.org/content/10.1101/2020.01.29.924266v2.full)	N/A
GTEx database	GTEx Consortium et al., 2020PMID: 32913098	N/A

**Software and algorithms**		

R v3.6.3	The R Project for Statistical Computing	https://www.r-project.org
BioRender	©BioRender	biorender.com

### Resource availability

#### Lead contact

Information and requests for resources should be directed to and will be fulfilled by the lead contact, Kuan-lin Huang (kuan-lin.huang@mssm.edu).

#### Materials availability

This study did not generate new unique reagents.

#### Data and code availability

All relevant data are available from the lead contact upon request. All code use in the eQTL Catalogue and colocalization analyses can be found in the following GitHub repository: https://github.com/RajLabMSSM/COVID19_PheWAS.

Additional Supplemental Items are available from Mendeley Data at http://doi.org/10.17632/nyn84yyms7.1 and https://data.mendeley.com/datasets/nyn84yyms7/1.

### Method details

#### Extraction of severe COVID-19 associated lead SNPs

We downloaded the summary statistics of the genome-wide meta-analysis that was corrected for age and sex as reported by The Severe Covid-19 GWAS group (Accession numbers: GCST90000256) (Ellinghaus et al., 2020). Aside from the reported SNPs, namely rs11385942 at locus 3p21.31 and with rs657152 at locus 9q34.2, we extracted the SNPs with suggestive associations (p < 1e-5), resulting in 319 SNPs from GCST90000256. We then pruned the associated SNPs based on the most significant p value per locus (±100Kb regions surrounding the lead SNP) recursively within the study, resulting in 22 non-adjacent loci, including 2 genome-wide significant SNPs (p < 5e-8) (Altshuler and Donnelly, 2005; Pe’er et al., 2008) and 20 suggestive SNPs associated with severe COVID-19 with respiratory failure (Table S1). We then cross-referenced 22 lead SNPs against the meta-analysis of GWAS studies between hospitalized COVID-19 patients and the population controls (B2_ALL, dated 09/30) performed by the COVID-19 Host Genetics Initiative (COVID-19 hg) (COVID-19 Host Genetics Initiative, 2020) We considered these SNPs’ association as “replicated” in the independent GWAS if they surpassed the Bonferroni-corrected p-value for the 22 queried SNPs (P < 0.05/22), validating associations for 3 additional SNPs (rs2440652, rs3934992, rs12610495) in addition to the two previously reported COVID-19 GWAS SNPs. In order to evaluate the level of independence of each SNP, linkage disequilibrium (LD) analysis was performed using the LDmatrix tool (Machiela and Chanock, 2015) (1) between any pair of the 22 lead SNPs that are located on the same chromosome ((Table S2) and (2) between each replicated SNP and other variants showing suggestive associations (p<1e-5) from GCST90000256 within ±100Kb surrounding each replicated SNP (Figure S1). Spanish and Italian population were used as reference population for the LDmatrix analysis to correspond with the population used in the original COVID-19 GWAS study.

#### PheWAS analysis

Phenotype associations of the 22 lead SNPs were queried using the PheWAS database available at the GWAS ATLAS(Watanabe et al., 2019) and the PheWAS results of each SNP was compiled into the candidate list of PheWAS hits (Table S3). Upon compilation, the list of associations was filtered using the genome-wide significance threshold (p < 5e-8) (Altshuler and Donnelly, 2005; Pe’er et al., 2008), resulting in 17 significant phenotypes. Considering the varying statistical thresholds used in the PheWAS literature(Denny et al., 2013; Diogo et al., 2018; Hebbring, 2014), we focused on a concise description of the associations passing the genome-wide significance threshold in the main texts, while noting that the full list of PheWAS results are available in Table S3 enabling examination of other associations.

#### Tissue- and immune-specific eQTL/sQTL analysis

Similar to the PheWAS analysis, we used our list of 22 lead SNPS to query tissue-specific expression QTL (eQTL) and splicing QTL (sQTL) datasets from the GTEx database(GTEx Consortium et al., 2020). In order to correct for multiple testing, e/sQTL associations were then filtered using the Benjamini-Hochberg(Benjamini and Hochberg, 1995), resulting in 258 eQTLs and 143 sQTLs with FDR<0.05. In addition to the GTEx analysis, we used the R package *catalogueR* (https://github.com/RajLabMSSM/catalogueR) (Schilder et al., 2020) to query these same 22 SNPs in the eQTL Catalogue(Kerimov et al., 2020), which contains 63 eQTL datasets across 20 studies (excluding GTEx). GWAS hits within sex chromosomes were excluded as the eQTL Catalogue only contains results for autosomes. eQTL Catalogue contains many immune subtype-specific and stimulation condition-specific datasets, permitting greater resolution of our biological interpretations. 190 significant eVariants associated with 9 lead GWAS SNPs (Table S6) remained after filtering by FDR<0.05 (Figure S2)—132 of which were immune subtype- or state- specific.

#### GWAS/eQTL colocalization analyses

Using the summary statistics of the original GWAS study (GCST90000256) and the eQTL Catalogue datasets, colocalization analysis was performed on 9 lead SNPs whose eVariants identified in the eQTL Catalogue were statistically significant (FDR<0.05). The locus for each lead SNP was defined as ± 1Mb from the lead SNP (2Mb windows). Since the original GWAS summary statistics do not provide both minor allele frequency (MAF) or the sample size for each SNP, MAF of each SNP was assumed to be the same as the one provided by the eQTL Catalogue. Each SNP was assumed to have the same effective sample size calculated from the total number of participants in the GWAS while accounting for the proportion of cases/controls using the formula:

$$\frac{4}{\left(\frac{1}{Ncases}+\frac{1}{Ncontrols}\right)}$$

A series of Bayesian colocalization tests of were run to evaluate the probability of shared genetic signal between each GWAS locus and each eQTL locus. Specifically, we used the *run_coloc()* function within *cataloguer* (Schilder et al., 2020)*,* which runs Approximate Bayes Factor colocalization (Wakefield, 2009) to first infer the probability that each SNP is causal for the phenotype within each respective dataset and then calculate the probability that the same underlying genetic signal is shared in both datasets. eQTL pseudogenes were identified via Ensembl and removed from downstream analysis. For each GWAS-QTL locus comparison, the signals were considered to be colocalized if they met the following criterion (where PP.H4 is the posterior probability that the signals are associated with their respective traits and shared, and PP.H3 is the posterior probability that the signals are associated with their respective traits but not shared):

$$\left(PP.H3\;+\;PP.H4\;>\;0.8\;\right)\;\&\;\left(\;\frac{PP.H4}{PP.H3}\right)\;\geq \;2$$

#### scRNA-seq analysis

Previously published single-cell RNA-seq (scRNA-seq) dataset (accession number: GSE145926) of bronchoalveolar lavage fluid (BALF) from COVID-19 patients and healthy controls was downloaded and analyzed for differential expression of six immune genes nominated from the immune-specific eQTL analyses above (*DPP9, NLRP1, GSDMD, CCR1, CCR2, CCR5)* in monocyte/macrophage subsets using the analytical pipeline described previously (Liao et al., 2020b) Briefly, using the cluster annotations described in the original publication to isolate monocyte/macrophage subsets, the macrophage clusters were then integrated using *IntegrateDat*a() function with default parameters from the Seurat v3 package (Stuart et al., 2019) and differential gene expression analysis between disease severity groups was performed using MAST (Finak et al., 2015) in Seurat v3(Stuart et al., 2019) to generate a list of differentially expressed genes between two groups (i.e. Severe vs. Moderate, Moderate vs. Healthy, Severe vs. Healthy). The list was then queried for specific immune genes of interest for their statistics. In accordance with Seurat's best practices, Bonferroni adjusted p values were computed taking into account the full number of genes tested in the dataset (Stuart et al., 2019). Violin plots comparing their expression levels as well as the UMAP plot of the integrated macrophage subset are shown in Figure S3.

### Quantification and statistical analysis

All quantification and statistical analyses were performed as described in the method details section of the STAR Methods.

## Supplemental information

Document S1. Figures S1–S3 and Table S2

Table S1. A list of pruned lead SNPs from GCST90000256, related to Figure 1

Table S3. Complete PheWAS hits associated with lead SNPs, related to Figure 1

Table S4. Complete list of eQTLs associated with lead SNPs compiled from GTEx database, related to Figure 2

Table S5. Complete list of sQTLs associated with lead SNPs compiled from GTEx database, related to Figure 2

Table S6. Complete list of QTLs associated with lead SNPs compiled from eQTL Catalogue, related to Figure 3
