# Supplementary material for: Phenome-wide and expression quantitative trait locus associations of coronavirus disease 2019 genetic risk loci
Source: iScience. 2021 May 18;24(6):102550. doi: 10.1016/j.isci.2021.102550 (PMC8129787; doi:10.1016/j.isci.2021.102550)
Supplement: Document S1. Figures S1–S3 and Table S2 [file mmc1.pdf]

iScience, Volume *24*

Supplemental information

Phenome-wide and expression quantitative trait locus associations of coronavirus disease 2019 genetic risk loci

Chang Yoon Moon, Brian M. Schilder, Towfique Raj, and Kuan-lin Huang

Supplementary Fig. 1

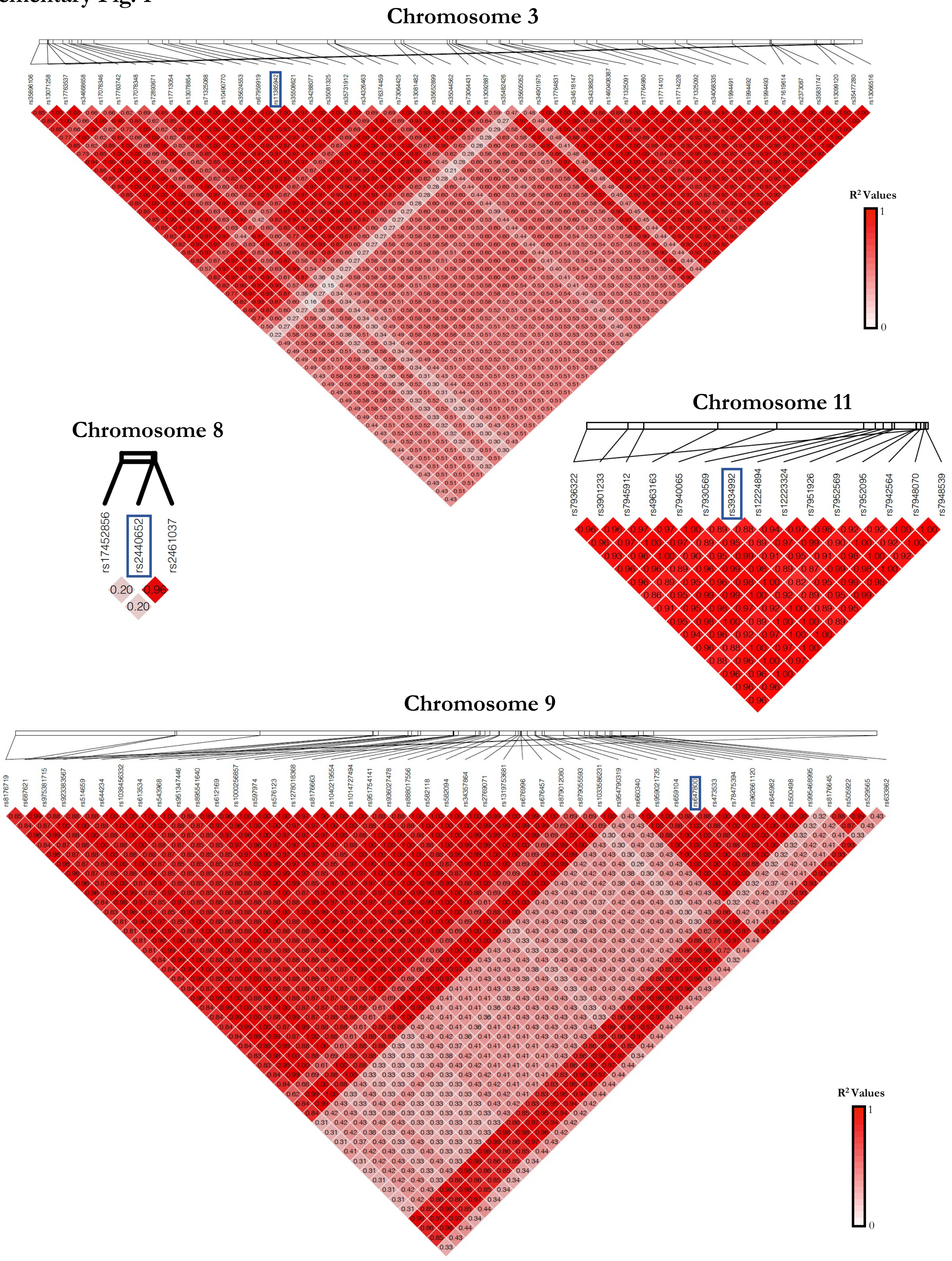

Supplementary Figure 1 (Related to Figure 1). LD analysis of each replicated SNP and its neighboring variants. The neighboring variants are defined as GWAS SNPs showing suggestive associations (P<1e-5) from GCST90000256 within ±100Kb surrounding each replicated SNP. Each square has a numeric value representing R^2 value of each pair of SNPs being tested and is characterized by red color scale with the brightest red representing R^2 value of 1. Each blue rectangle indicates each replicated SNP. A replicated SNP at chromosome 19 (rs12610495) is not shown as there were no neighboring variants (P<1e-5) within the ±100Kb window

Supplementary Fig. 2

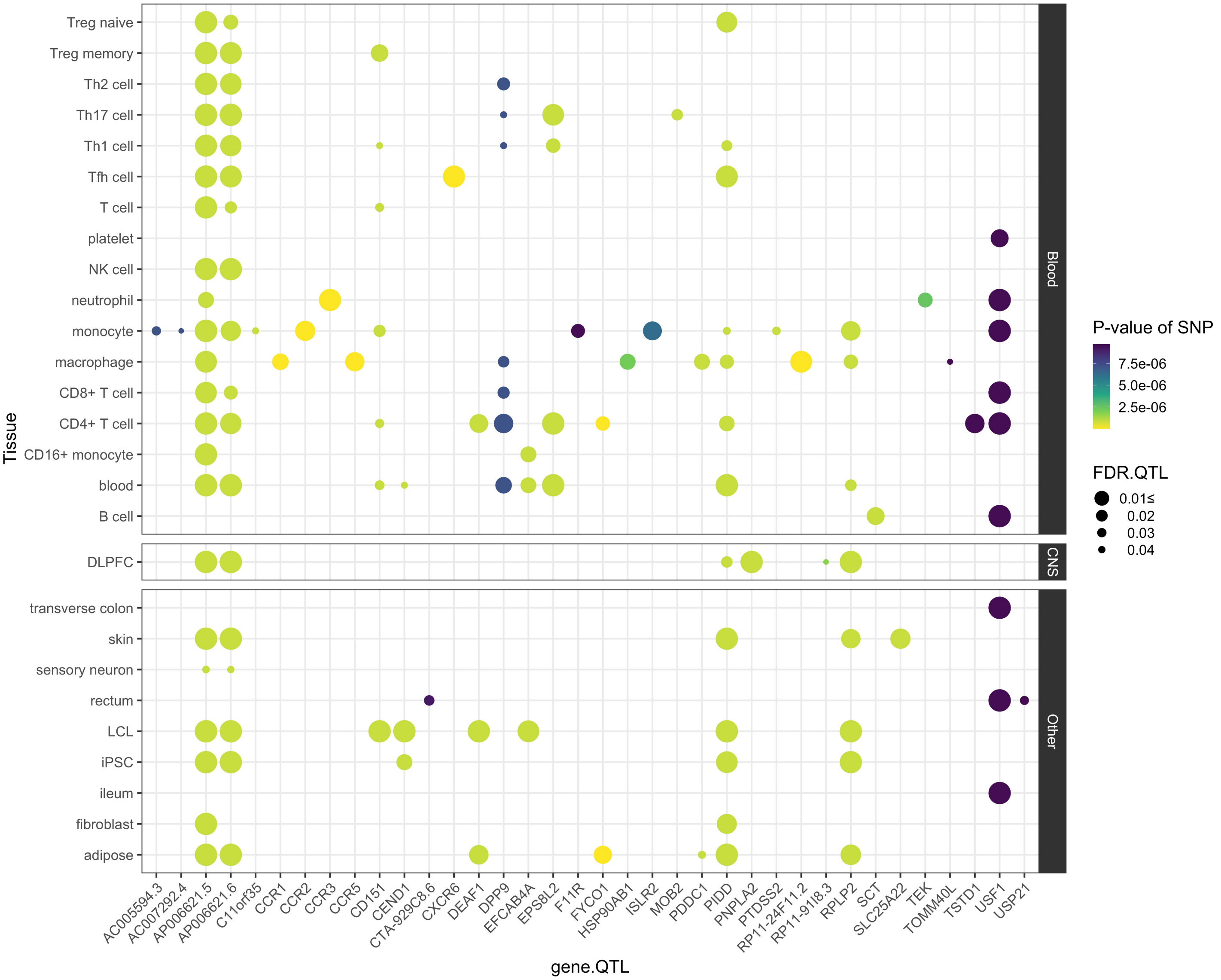

Supplementary Figure 2 (Related to Figure 3). Significant Immune/Non-immune specific eQTLs associated with lead SNPs. The eQTLs were compiled from the eQTL Catalogue[13] database and filtered at a Benjamini-Hochberg FDR < 0.05. Point color reflects the significance of that SNP in the COVID-19 GWAS[6] (more yellow = more significant p-values), while point size reflects the significance of the eQTL SNP (larger = more significant FDR). When multiple SNPs overlap within a given gene-tissue combination, the SNP with the lowest p-value/FDR is plotted.

Supplementary Fig. 3

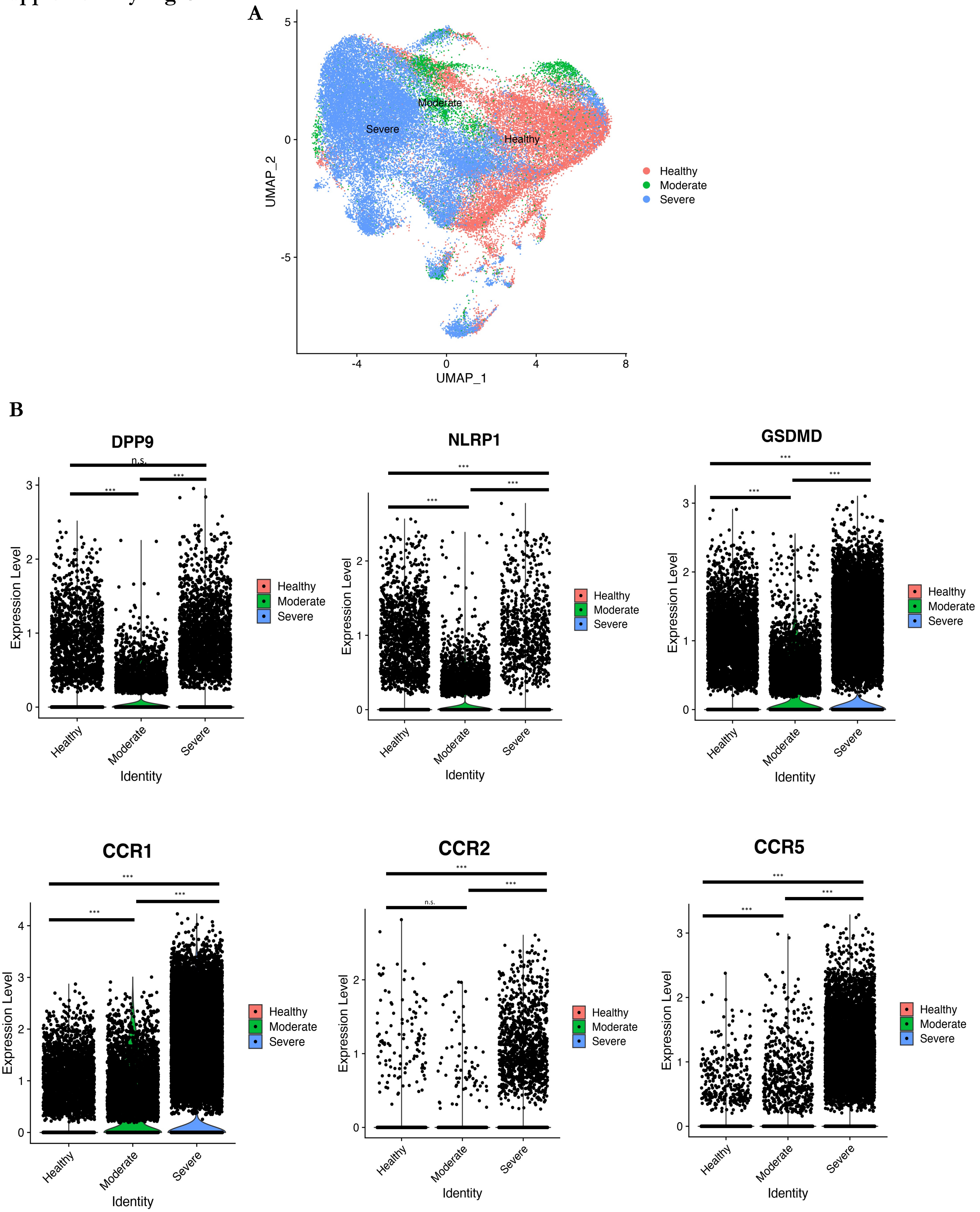

Supplementary Figure 3 (Related to Figure 3). scRNA-seq of Macrophages From Bronchoalveolar Lavage Fluid of COVID-19 Patients. A) UMAP plot of integrated macrophage subset from scRNA-seq of bronchoalveolar lavage fluid from COVID-19 patients and controls. Each disease severity is denoted with a color. B) Differential expression of immune genes between monocytes/macrophages of different disease severity groups based on scRNA-seq of bronchoalveolar lavage fluid from COVID-19 patients and healthy controls. *** indicates Bonferroni adjusted P < 0.001 and n.s. indicates non-significant.

Supplementary Table 2 (Related to Figure 1). LD statistics of intra-chromosome lead SNPs

LD Pairwise Statistics	D'	R^2
chr1 (rs114173811; rs75558547)	1	0.001
chr3 (rs9882319; rs11385942)	0.085	0
chr8 (rs76670680; rs2440652)	0.34	0.003
chr9 (rs576557083; rs647800)	0.286	0.002
chr11 (rs3934992; rs78243302)	0.238	0.001
chrX (rs62586074; rs113076449)	1	0
chrX (rs62586074; rs10126492)	0.363	0.001
chrX (rs113076449; rs10126492)	1	0.006
